# Arbuscular Mycorrhizal Fungus Alters Root System Architecture in *Camellia sinensis* L. as Revealed by RNA-Seq Analysis

**DOI:** 10.3389/fpls.2021.777357

**Published:** 2021-11-12

**Authors:** Weili Chen, Tao Ye, Qinyu Sun, Tingting Niu, Jiaxia Zhang

**Affiliations:** Tea Research Institute, Anhui Academy of Agricultural Sciences, Huangshan, China

**Keywords:** arbuscular mycorrhizal fungus, tea plant, root branching, phytohormones, phosphorus, sugar, lipid

## Abstract

Arbuscular mycorrhizal fungus (AMF), forming symbiosis with most terrestrial plants, strongly modulates root system architecture (RSA), which is the main characteristic of root in soil, to improve plant growth and development. So far, the studies of AMF on tea plant seedlings are few and the relevant molecular mechanism is not deciphered. In this study, the 6-month-old cutting seedlings of tea plant cultivar “Wancha No.4” were inoculated with an AMF isolate, *Rhizophagus intraradices* BGC JX04B and harvested after 6 months of growth. The indexes of RSA and sugar contents in root were determined. The transcriptome data in root tips of mycorrhizal and non-mycorrhizal cutting seedlings were obtained by RNA-sequence (Seq) analysis. The results showed that AMF significantly decreased plant growth, but increased the sucrose content in root and the higher classes of lateral root (LR) formation (third and fourth LR). We identified 2047 differentially expressed genes (DEGs) based on the transcriptome data, and DEGs involved in metabolisms of phosphorus (42 DEGs), sugar (39), lipid (67), and plant hormones (39) were excavated out. Variation partitioning analysis showed all these four categories modulated the RSA. In phosphorus (P) metabolism, the phosphate transport and release (DEGs related to purple acid phosphatase) were promoted by AMF inoculation, while DEGs of sugar transport protein in sugar metabolism were downregulated. Lipid metabolism might not be responsible for root branching but for AMF propagation. With respect to phytohormones, DEGs of auxin (13), ethylene (14), and abscisic acid (5) were extensively affected by AMF inoculation, especially for auxin and ethylene. The further partial least squares structural equation modeling analysis indicated that pathways of P metabolism and auxin, as well as the direct way of AMF inoculation, were of the most important in AMF promoting root branching, while ethylene performed a negative role. Overall, our data revealed the alterations of genome-wide gene expression in tea plant roots after inoculation with AMF and provided a molecular basis for the regulatory mechanism of RSA (mainly root branching) changes induced by AMF.

## Introduction

The root plays a great important role in plant growth and development through absorbing mineral nutrients and water, anchoring the aboveground, storing carbohydrates and lipids, and secreting an enormous range of compounds with growth regulatory properties. The root trait in the soil is mainly characterized as root system architecture (RSA), namely the spatial configuration of root system covering root length, root branching, and root diameter (Lynch, 1995). Among all the ingredients in RSA, root branching, including adventitious root (AR) and LR formation, is one of the most important to define the function of promoting plant growth. Excellent RSA contributes to adaptation to various adverse environments, making it possible that plants will grow healthily and effectively (Chen et al., 2016). For instance, plant roots with more branching, coming with more root tips and foraging area, could grow well in phosphorus-deficient soils through a citrate-enhanced uptake way (McKay Fletcher et al., 2020). In turn, RSA, showing great plasticity, is influenced by soil water and nutrient, soil texture, heterogeneity, and biotic interaction (Balliu et al., 2021; Lombardi et al., 2021), wherein arbuscular mycorrhizal fungus (AMF), which naturally forms a symbiosis with about 80% terrestrial plant species, could affect RSA by enhancing LR formation through the auxin pathway (Chen et al., 2017a). The successfully constructed mycorrhiza will not only promote plant growth, but also elevate abiotic stress tolerance of most plants, including drought, water logging, low temperature, salinity, and so on, which was intensively reviewed by Begum et al. (2019).

Tea plant (*Camellia sinensis* L.) originated from China has become the most prevalent non-alcoholic beverage around the world. With the increase of planting area, various problems in the tea industry have arisen. Many abiotic (drought, waterlogging, high temperature, low temperature, etc.) and biotic stress (diseases, pests, weeds, etc.) seriously restrict the high-yield and superior-quality production of tea, particularly in tea garden composed of clonal cultivars with the shallow root system (Chen et al., 2020). Mycorrhizal symbiosis was first found in tea plant rhizosphere in the early twentieth century, and tea plants show the strong mycorrhizal dependency (Gao et al., 2011). Liu et al. (2021a) recently reviewed that AMF plays great importance in *Camellia* spp., such as promoting growth and development, absorbing nutrients and obtaining abilities of resistance to drought, heavy metal, salt stress, etc. For instance, an array of studies indicated that inoculation with AMF improves leaf qualities (Shao et al., 2019) and enhances tea plant’s tolerance with salinity (Liu et al., 2013; Xia and Wen, 2017), in which AMF could positively modify the RSA, like root length, LR formation, and root biomass (Sharma and Kayang, 2017; Shao et al., 2018), to promote plant growth and development. However, the researches about the modulation of AMF on tea plant RSA are a few and many only focused on physiological and biochemical levels with the internal mechanism not deciphered. Thus, in this article, we conducted a pot experiment to observe the effect of AMF on RSA in tea plant cutting seedlings of 6 months old and unravel the corresponding molecular mechanisms by RNA-sequence (Seq), providing a theoretical basis for further research on tea plant root system.

## Materials and Methods

### Experimental Materials and Design

The 6-month-old “Wancha No.4” (Wang et al., 2018) cutting seedlings with about 5 cm height were used as tea plant (*Camellia sinensis* L.) materials, which were inoculated with AMF in pot culture, and AMF isolate was *Rhizophagus intraradices* BGC JX04B provided by Beijing Academy of Agriculture and Forestry Sciences (Chen et al., 2017a). *R. intraradices* is widely used in the study of the interaction between plants and AMF, which can colonize the root of most mycorrhizal plants well. The inocula, obtained by propagating the isolate with clover (*Trifolium repense* L.) as hosts for 3 months in the greenhouse, were ca. 40 spores per gram in spore density. The growth substrate was the mixture of autoclaved (121°C, 2 h) soils and peat (1:1, v:v). The soils were collected from the tea plant garden of Tea Research Institute, Anhui Academy of Agricultural Sciences, Huangshan, China (29°41′18″E, 118°15′33″N), and the soil chemical properties were determined as follows: pH 5.13, organic matter content 5.17 g⋅kg^–1^, available nitrogen (N) 31.40 mg⋅kg^–1^, available phosphorus (P) 2.52 mg⋅kg^–1^, and available potassium (K) 76.20 mg⋅kg^–1^. The substrate was additionally applied with 200 mg⋅kg^–1^ N ((NH_4_)_2_SO_4_), 50 mg⋅kg^–1^ P (KH_2_PO_4_), and 100 mg⋅kg^–1^ K (KNO_3_).

A pot experiment with two treatments [non-mycorrhizal (C) and mycorrhizal (T)] was set up to reveal the effect of AMF on RSA of tea plant cutting seedlings. Each pot with one tea plant cutting seedling represented one biological replicate, and three replicates, namely three pots, were set up in each treatment. Each pot was added with 950 g substrate and 50 g AMF inocula (T treatment) or sterilized inocula (C treatment). To avoid the potential influence of other microbes, we also added 5 mL filtrates (25-μm filter) of AM fungal inoculum to the C treatment. All mycorrhizal and non-mycorrhizal seedlings grew in the greenhouse under natural light conditions with 22∼30°C and 70∼80% relative humidity. Plants were harvested at 6 months after transplanting.

### Sampling and Mycorrhizal Colonization Detection

After total seedlings were gently washed out from the soil, shoots and roots were separated and the fresh weights were recorded, respectively. The ScanMaker i800 Plus scanner (Hangzhou WSeen Detection Technology Co., Ltd., China) was used to obtain root system pictures with high resolution, followed by quantifying the indexes of RSA by LA-S (Leaf and Root Analysis System) plant image analyzer system (Hangzhou WSeen Detection Technology Co., Ltd., China). The scanning process must be accomplished quickly to avoid the interior change in roots. Then, the root tips (about 1.5 cm length) were randomly cut down (about 0.2 g) and preserved at −80°C for RNA-seq and quantitative real-time PCR (qRT-PCR) analysis. The rest of the root was sectioned into fragments with about 1 cm length and 50 fragments were randomly picked up for the measurement of mycorrhizal colonization. Other fragments were used for carbohydrate determination. Mycorrhizal staining was performed according to Phillips and Hayman (1970). Specifically, the root segments were incubated with 10% KOH (w/v) in a water bath at 60°C for 60 min, then rinsed with tap water, bleached with alkaline hydrogen peroxide (10% H_2_O_2_ + NH_4_OH) for 15 min, acidified in 2% HCl solution for 5 min, and stained with 0.05% Trypan blue in lactoglycerol (lactic acid:glycerol: water, v:v:v = 1:1:1) at 60°C for 30 min. After decolorizing in lactic acid: glycerol (v:v = 1:1) for 24 h, each root segment was placed onto a slide and estimated under an optical microscope (OLYMPUS BX51), and then the mycorrhizal colonization was quantified according to Biermann and Linderman (1981).

### Physio-Biochemical Parameters Determination

After being digested with HNO_3_, the shoot P contents were measured by a spectrophotometer according to Arshad et al. (2016). The carbohydrate contents (sucrose, reducing sugar, and soluble sugar) in fresh roots were colorimetrically determined according to Xue (1985) and Gao (2006) with some modifications. Briefly, 0.2 g fresh roots were homogenized with 4 mL 80% ethanol, incubated for 30 min at 90°C, and then centrifuged at 8,000 rpm for 10 min. The residues were extracted two times as described above, and all supernatants were combined for subsequent analysis. Total soluble sugar was assayed with a mixture of 40 μL supernatant, 160 μL deionized water, and 3 mL 0.2% anthrone in 95% sulfuric acid at 90°C for 15 min followed by measurement of absorbance at 620 nm. The sucrose content was determined by mixing 40 μL supernatant, 160 μL deionized water, and 100 μL 30% KOH at 90°C for 10 min, then adding 3 mL anthranone and bathing at 45°C for 30 min. After cooling, the OD_620_ (optical density) was measured. Reducing sugar was assayed with a mixture of 0.3 mL supernatant, 1.7 mL deionized water, and 1.7 mL dinitrosalicylic (DNS) acid at 90°C for 5 min followed by measurement of OD_520_. The carbohydrate contents were quantified according to the linear standard curves constructed with respective sugars.

### RNA-Seq, Transcriptomic Analysis, and qRT-PCR

The total RNA in the root tips from C and T treatments (three independent biological replicates in each treatment) was isolated with an RNA extraction kit (Accurate Biotechnology Co., Ltd., Hunan, China) according to the manufacturer’s protocol. The RNA integrity was checked with Agilent 2100 Bioanalyzer and Agilent RNA 6000 Nano Kit, and RNA concentration was determined using Denovix DS-11 spectrophotometer (Denovix Inc., United States). One aliquot was sent to Tsingke Biotechnology Co., Ltd. (Beijing, China) for RNA-seq by using the Illumina Hiseq™ 4,000 platform and 150 bp paired-end reads were generated. After removing reads containing adapters or more than 50% N and low-quality reads from the raw reads, clean reads were obtained for all subsequent analyses. The *Camellia sinensis* reference genome (Wei et al., 2018), directly downloaded from the Tea Plant Information Archive (TPIA),^1^ was used for the mapping of clean reads by HISAT (Kim et al., 2015). For ascertaining the differentially expressed genes (DEGs) between C and T treatments, after aligning clean reads to reference sequences by Bowtie2 (Langmead and Salzberg, 2012) and calculating the expression levels of genes by StringTie (Pertea et al., 2015), DESeq2 (Love et al., 2014) was performed with *Q*-value ≤ 0.05. Gene ontology (GO) enrichment analysis of DEGs was implemented by the Blast2GO software (Conesa et al., 2005). Based on Kyoto encyclopedia of genes and genomes (KEGG),^2^ the KOBAS 2.0 software^3^ was used to test the statistical enrichment of DEG in KEGG pathways (Xie et al., 2011).

### Quantitative Real-Time-PCR Analysis

Another aliquot of extracted RNA was used for qRT-PCR according to the previous protocol (Chen et al., 2017a). *18s rRNA* (AB120309.1) was used as a reference gene for qRT-PCR analysis in tea plants (Wei et al., 2013). All primers (Supplementary Table 1) of randomly selected genes, designed by using Primer 3 software to amplify 150–200 bp fragments, were synthesized in Tsingke Biotechnology Co., Ltd (Beijing, China). The relative expression of each target gene was calculated by using 2^–ΔΔCt^ method (Livak and Schmittgen, 2001).

### Statistical Analysis

All data were presented as mean ± SE of three replicates. The *t*-test was performed and the correlations between the indexes of RSAs and DEGs were calculated with bivariate correlations analysis using SPSS (Statistical Package for the Social Sciences) v.25 statistical software (SPSS Inc., Chicago, IL).

The variation partitioning analysis (Peres-Neto et al., 2006) based on redundancy analysis (RDA) was performed to quantify the contributions of different categories of DEGs to the construction of RSA in tea plant cutting seedlings. Four explanatory variables (DEGs related to metabolisms of phosphorus, sugar, plant hormone, and lipid, respectively) were conducted using “varpart” function in R vegan package (Oksanen et al., 2013), and the generated fractions (appeared as percentage) of variation were explained by different variables with or without covariable. Normally, the explanatory variable with a higher percentage contributes more greatly to the response variable than the other explanatory variable with a lower percentage (Peres-Neto et al., 2006).

To find the key genes in AM fungal regulation on RSA, the correlations between DEGs and root indexes were discovered by performing RDA and visualized in CANOCO 5 software (Braak and Smilauer, 2002). Additionally, according to previous studies (Fusconi, 2014; Chen et al., 2017a), we proposed a hypothetical model, which was specified and analyzed with Partial Least Squares Structural Equation Modeling (PLS-SEM) with the support of WarpPLS (version 6.0) software (Kock, 2017), to explore the relationships between AMF inoculation, root branching, and metabolisms of phosphorus, sugar, plant hormones, and lipid.

## Results

### Mycorrhizal Colonization and Plant Growth Status

As shown in Table 1, mycorrhizal colonization was 26.67% in T treatment, while that was not detected in the C treatment. After AMF inoculation, plant growth was slowing. The plant height, fresh weight of shoot, and root were significantly lower than those of the C treatment, while the R/S (root/shoot) ratio was slightly lower in T treatment.

**TABLE 1:** Results of plant biomass and relevant indexes of root system architecture induced by AMF inoculation.

Treatment	Mycorrhizal colonization and plant biomass				
	Colonization (%)	Plant Hight (cm)	Shoot FW(g)	Root FW(g)	R/S
C	0.00 ± 0.00	21.44 ± 0.93*	4.38 ± 0.33*	2.06 ± 0.19*	0.47 ± 0.01
T	26.67 ± 1.20***	13.95 ± 1.53	3.42 ± 0.08	1.50 ± 0.02	0.44 ± 0.01

	**General indexes of root system architecture**				
	**TRL (cm)**	**TPA (cm^2^)**	**TSA (cm^2^)**	**TV (cm^3^)**	**AD (mm)**

C	782.47 ± 30.57	34.69 ± 2.15	108.98 ± 6.77	1.77 ± 0.11	0.82 ± 0.15*
T	648.09 ± 35.48	28.58 ± 1.18	89.79 ± 3.71	1.48 ± 0.06	0.48 ± 0.02

“*” and “***” indicated significance highlighted in bold font at p < 0.05 and p < 0.001 in each column, respectively; FW, fresh weight; R/S, root biomass/shoot biomass. TRL, total root length; TPA, total root projected area; TSA, total root surface area; TV, total root volume; AD, average root diameter; C, non-mycorrhizal treatment; T, mycorrhizal treatment. All values were mean value ± SE of three biological replicates.

**FIGURE 1:**
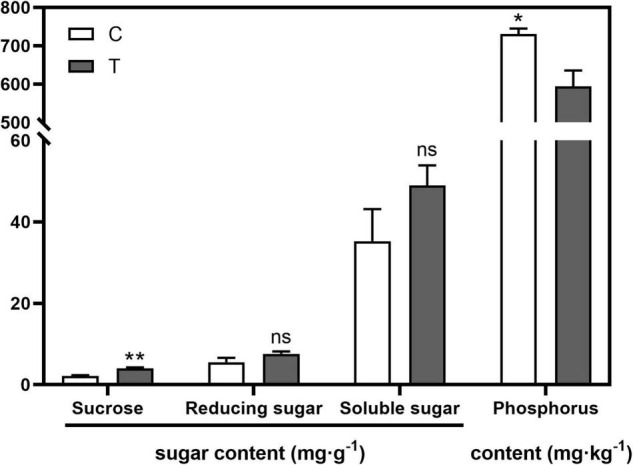
The influences of arbuscular mycorrhizal fungus (AMF) on contents of sugar in tea plant root and phosphorus in shoot. “*” and “**” mean significant difference at *p* < 0.05 and *p* < 0.01, respectively. “ns” was short for “no significance.”

AMF inoculation significantly increased sucrose content (*p* < 0.01) in the root compared with the C treatment, while no significant difference was found in the contents of reducing sugar and soluble sugar (Figure 1). With respect to the phosphorus content in the aboveground part, AMF significantly decreased it (594.43 mg⋅g^–1^, *p* < 0.05), over 100 mg⋅g^–1^ less than that in the C treatment (731.00 mg⋅g^–1^, Figure 1).

### Alterations in Tea Plant Root System Architecture by Arbuscular Mycorrhizal Fungus Inoculation

**FIGURE 2:**
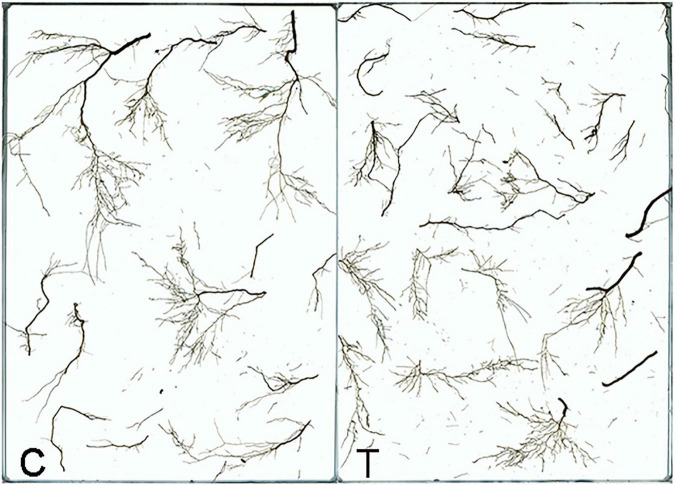
The root system of tea plant seedlings as affected by AMF. C: non-mycorrhizal treatment, T: mycorrhizal treatment.

Overall, AMF inoculation decreased the root morphogenesis, including total root length (TRL), total root projected area (TPA), total root surface area (TSA), total root volume (TV), and total length of roots with a different diameter (Figure 2 and Tables 1, 2), but not reaching significant level. Moreover, in the mycorrhizal treatment, the average root diameter was significantly reduced, almost two times less than that in the non-mycorrhizal treatment (Table 1).

**TABLE 2:** Alterations in root length of each diameter induced by AMF inoculation.

Treatment	Total length of different classes (cm)			
	0.000 ≤ D < 0.500	0.500 ≤ D < 2.000	2.000 ≤ D < 3.000	3.000 ≤ D < 5.000
C	592.35 ± 6.97	185.32 ± 26.40	4.46 ± 1.53	0.34 ± 0.29
T	486.29 ± 41.31	158.01 ± 16.06	3.74 ± 1.58	0.04 ± 0.04

*“D” indicated “diameter of root.” All values were mean value ± SE of three replicates.*

### Root Branching as Affected by Arbuscular Mycorrhizal Fungus Inoculation

The AR and LR formation were greatly induced by AMF inoculation, except for the first and second LR (Table 3). Since the overall increase of the root biomass in the control, the second LR number, second LR/first LR ratio, total LR (TLR) number were significantly increased compared with the mycorrhizal treatment, as well as the first LR number and first LR/AR ratio but with no significant difference (Table 3). In addition to the slight increase of AR and third LR number, inoculating with AMF significantly promoted the higher classes of LR formation. Specifically, the fourth LR was only found in the mycorrhizal treatment, and ratios of third LR/second LR, fourth LR/third LR, third LR/TRL, and fourth LR/TRL were significantly elevated, indicating more formation capacity of LR in contrast to the control.

**TABLE 3:** Statistical results of root numbers and densities at all classes induced by inoculating with AMF.

Treatment	Root number								
	AR	1st LR	2nd LR	3rd LR	4th LR	TLR		
C	8.67 ± 0.33	147.67 ± 8.88	205.67 ± 16.01*	25.00 ± 6.11	0.00 ± 0.00	387.00 ± 31.21*		
T	10.33 ± 1.20	139.00 ± 11.93	121.67 ± 14.81	35.33 ± 5.49	1.67 ± 0.67***	308.00 ± 16.09		

	**Root density**								
	**1st LR/AR**	**2nd LR/1st LR**	**3rd LR/2nd LR**	**4th LR/3rd LR**	**1st LR/TRL**	**2nd LR/TRL**	**3rd LR/TRL**	**4th LR/TRL**	**Total LR/TRL**

C	17.01 ± 0.39	1.39 ± 0.03*	0.12 ± 0.02	0.00 ± 0.00	18.87 ± 0.68	26.22 ± 1.13	0.02 ± 0.00	0.000 ± 0.000	0.49 ± 0.02
T	13.85 ± 2.08	0.88 ± 0.10	0.31 ± 0.08**	0.04 ± 0.01*	21.85 ± 3.24	18.95 ± 2.55	0.05 ± 0.01*	0.002 ± 0.000*	0.48 ± 0.05

AR, adventitious root; LR, lateral root; TLR, total lateral root; TRL, total root length; For each column, values (mean value ± SE of three replicates) followed by “*,” “**,” and “***” mean significant difference highlighted in bold font at p < 0.05, p < 0.01 and p < 0.001, respectively.

### Bioinformatics Analysis of RNA-Seq Data and Identification of Differentially Expressed Genes in Roots

RNA-seq analysis was simultaneously performed to reveal the molecular mechanisms possibly involved in the AM Fungal regulation on the RSA of the tea plant cutting seedlings. In Table 4, overview results of RNA-seq were outlined. After trimming and applying a quality filter, clean reads were obtained and the proportion of clean Q30 bases were all over 91% (Table 4). Over 81% of these clean reads for each biological repeat mapped into the *Camellia sinensis* genome in order to find DEGs between the mycorrhizal and non-mycorrhizal treatment. And the RNA-seq dataset, used for analysis in this article, was deposited in National Center for Biotechnology Information (NCBI) Short Read Archive (SRA)^4^ under the BioProject of PRJNA763027.

**TABLE 4:** Main results of RNA-Seq.

Sample	Clean reads (M)	Clean Q30 bases (%)	GC (%)	Mapped reads	Mapped unique reads	Mapping ratio (%)
C1	45.9824	91.72	45.37	37385172	34960842	81.30
C2	44.6833	92.08	45.37	36918974	34299454	82.62
C3	39.6539	91.51	45.5	32540662	30441510	82.06
T1	44.7332	92.19	45.61	36999854	34033848	82.71
T2	46.1800	92.16	46.85	38586618	33659310	83.56
T3	44.6340	92.39	45.8	37107944	34117202	83.14

*C: non-mycorrhizal treatment; T: mycorrhizal treatment.*

**FIGURE 3:**
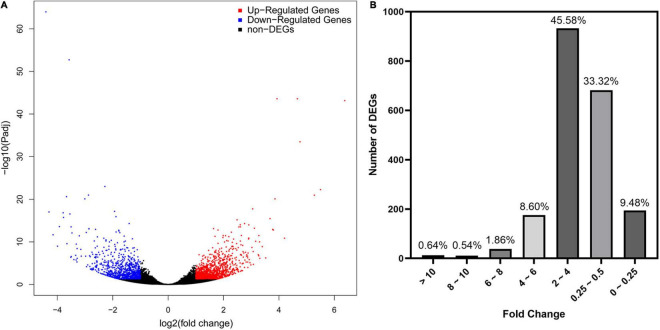
Preliminary statistics of differentially expressed genes (DEGs) between mycorrhizal and non-mycorrhizal treatment. **(A)**: Differentially expressed genes in volcano plot. Blue and red dots indicate the significantly downregulated and upregulated genes, respectively. **(B)**: Number of DEGs grouped by fold change. “Fold Change” indicates the ratio of expressions in T and C treatment of the same gene.

As revealed by RNA-seq, 2047 genes were significantly expressed induced by AMF inoculation, in which 1171 genes and 876 genes were up and downregulated, respectively (Figure 3A). Most DEGs were distributed in the range of 2∼4 (933, 45.58%) and 0.25∼0.5 (682, 33.32%) fold showed in Figure 3B. The upregulated DEGs by inoculating AMF was account for 57.12% of the total number. The most 10 up and downregulated DEGs were listed in Table 6 after removing those without accurate annotations. It is noteworthy that genes related to terpenoid synthase expressed significantly higher by 38.772 or 18.398 times in the mycorrhizal treatment (Table 6). We also found that *pectinesterase 2* (114257568) was more than 10 times upregulated by AMF inoculation compared with the C treatment, which might promote the entrance of AMF into the cell layers and subsequent symbiosis establishment, indirectly indicating the successful inoculation.

**TABLE 5:** The effects of explanatory variables on the indexes of root system architecture of tea plant cutting seedlings *via* variation partitioning analysis.

Response variable: indexes of root system architecture	df	Fraction explained (%)
**Explanatory variables**		
A fraction (*with covariable B, C, D*)	1	12.41
B fraction (*with covariable A, C, D*)	1	9.43
C fraction (*with covariable A, B, D*)	1	12.69
D fraction (*with covariable A, B, C*)	1	19.69
Total fraction	4	19.72
A fraction (*without covariable*)	1	21.16
B fraction (*without covariable*)	1	0.67
C fraction (*without covariable*)	1	−10.73
D fraction (*without covariable*)	1	45.44
Residuals	−	80.29

*All fractions (shown as percentages) explained were significant (p < 0.05). A, DEGs of Phosphorus metabolism; B, DEGs of Sugar metabolism; C, DEGs of Plant hormone metabolism; D, DEGs of Lipid metabolism.*

**TABLE 6:** The most 10 upregulated and downregulated DEGs between the mycorrhizal and non-mycorrhizal treatment.

Upregulated DEGs			Downregulated DEGs		
Gene ID	Fold change	Gene annotation	Gene ID	Fold change	Gene annotation
114288835	38.772	Terpenoid synthase, partial	114267754	0.047	Probable xyloglucan endotransglucosylase/hydrolase protein 23
114319070	25.370	Ferric-chelate reductase	114273524	0.051	Lactate dehydrogenase
114282497	18.398	Bifunctional terpenoid synthase	114256184	0.056	Protein NRT1/PTR FAMILY 7.3-lik
114306040	15.279	Metal transporter Nramp5-like	114263304	0.066	Putative dehydrin
114322081	13.933	Aquaporin TIP1-2-like	114316147	0.072	Type I inositol 1,4,5-trisphosphate 5-phosphatase 2 isoform X2
114277111	13.695	High affinity nitrate transporter 2.5	114280424	0.073	L-ascorbate oxidase homolog
114257568	11.516	Pectinesterase 2	114296016	0.084	Neurofilament medium polypeptide-like
114301937	10.549	1-deoxyxylulose 5-phosphate synthase	114323882	0.088	Ethylene-responsive transcription factor ERF110
114285402	9.923	2-oxoglutarate-dependent dioxygenase AOP2-like	114317784	0.089	Thaumatin-like protein
114314656	9.682	Calcium-binding protein KIC	114263353	0.099	Sugar transport protein 1-like

### Validation of Sequencing Results Through qRT-PCR

To validate the RNA-seq data, 12 genes were randomly selected for expression analysis *via* qRT-PCR. The expressions of these genes identified by qRT-PCR were as the same trend as observed in RNA-seq (Supplementary Figure 1), and there was a significantly positive correlation (*R*^2^ = 0.9387, Supplementary Figure 2) between RNA-seq and qRT-PCR data, indicating the RNA-seq data were credible for exploring the mechanism of phenotypic changes of root system induced by AMF inoculation.

### Functional Classification of Differentially Expressed Genes

All DEGs were assigned to GO categories and we found 42 significant GO terms, containing 20 DEGs in biological process, 13 DEGs in a cellular component, and 9 DEGs in molecular function. GO terms of each category based on the number of DEGs were shown in Supplementary Figure 3 and Supplementary Data 3. In detail, in the category of biological process, metabolic process (697 DEGs), cellular process (568 DEGs), localization (174 DEGs), response to stimulus (166 DEGs), and biological regulation (154 DEGs) were strongly highlighted. In the cellular component category, GO terms related to cell development were enriched with more DEGs, such as 528 DEGs in the membrane, 406 DEGs in membrane part, 399 DEGs in cell, and 394 DEGs in cell part. GO terms of catalytic activity (765 DEGs), binding (544 DEGs), and transporter activity (128 DEGs) in the molecular function category were enriched with more than 100 DEGs.

Meanwhile, we also conducted the KEGG pathway analysis and the top 20 KEGG pathways, based on RichFactor, were demonstrated in Supplementary Figure 4 and Supplementary Data 3. The pathways of metabolic pathways and biosynthesis of secondary metabolites were enriched with 401 and 260 DEGs, respectively, followed by the phenylpropanoid biosynthesis pathway (84 DEGs). Additionally, a total number of 45 DEGs belonged to fatty acid metabolism.

### Differentially Expressed Genes Pertaining to Metabolisms of Plant Hormone, Phosphorus, Sugar, and Lipid

**FIGURE 4:**
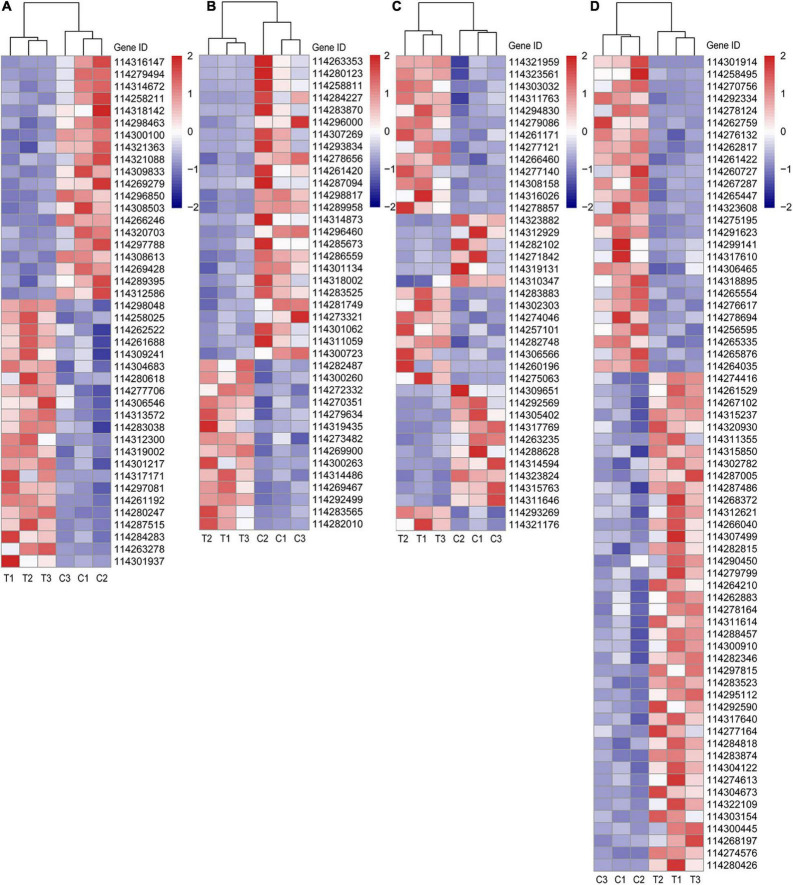
Different categories of DEGs. **(A)** DEGs related to P metabolism; **(B)** DEGs related to sugar metabolism; **(C)** DEGs related to plant hormone; **(D)** DEGs related to lipid metabolism. C: non-mycorrhizal treatment, T: mycorrhizal treatment. The annotation of DEGs is listed in Supplementary Data 2.

Taking the close relationship between RSA construction and P, sugar supply and plant hormone (especially auxin) into consideration, we selected the DEGs related to metabolisms of P, sugar, and plant hormone based on the gene description, namely “Blast nr” in Supplementary Data 2. Furthermore, since lipid metabolism induced by AMF is becoming a research hotspot on account of the deficient lipid biosynthesis in mycorrhiza together with the results of KEGG enrichment analysis in this article, we also selected lipid-relative DEGs. All these DEGs of different categories were visualized in Figure 4 and more corresponding information can be found in Supplementary Data 2.

In Figure 4A, the up (22 DEGs) and downregulated (20 DEGs) DEGs of P metabolism affected by AMF inoculation were account for about a half, respectively. Four DEGs (*114304683*, *114283038*, *114301217*, and *114261192*), described as purple acid phosphatase, were both upregulated by inoculating AMF. Noteworthily, compared with the C treatment, the expression of *phosphate transporter PHO1 homolog 1* (Gene ID: *114261688*) in tea plant root tips was nearly two times higher under the T treatment. Only *114301937* (*1-deoxyxylulose 5-phosphate synthase*) was expressed over 10 times in mycorrhizal treatment more than that in the C treatment, while the expressions of *114279494* (*type I inositol 1,4,5-trisphosphate 5-phosphatase 2 isoform X2*) and *114316147* (*type I inositol 1,4,5-trisphosphate 5-phosphatase 2 isoform X2*) were elevated in the C treatment, exceeding 10 times compared with the AMF inoculation. In addition, *soluble inorganic pyrophosphatase* (*114300100*) was also downregulated by AMF. With respect to sugar metabolism, the expressions of 25 out of 39 DEGs were lower in T treatment than those in C treatment (Figure 4B). Interestingly, all the four DEGs pertaining to sugar transport protein (*114263353*, *114280123*, *114287094*, and *114314873*) were significantly expressed lower induced by AMF inoculation than those in the control. Same results were also found in genes related to carbohydrate esterase (*114296460*, *114281749*, and *114273321*). For DEGs belonging to plant hormone metabolism, 23 out of 39 were upregulated after AMF inoculation (Figure 4C). It is notable that all auxin-related genes (13 DEGs) were upregulated in the mycorrhizal treatment, as well as *114321176* (related to salicylic acid), while DEGs, related to cytokinin (2 DEGs), gibberellin (3 DEGs), and abscisic acid (ABA, 5 DEGs), were downregulated, except for one ABA-related gene (*114293269*), which is described as *abscisic acid-responsive (TB2/DP1, HVA22) family protein*. Moreover, there were 14 DEGs connected to ethylene with eight expressing higher in the mycorrhizal treatment. Seven out of 12 DEGs related to the ethylene responsive factor were upregulated by AMF inoculation, indicating various genes’ function ways are not in the same way. As shown in Figure 4D, a total number of 67 DEGs were assigned to lipid metabolism, wherein 41 were upregulated and 26 were downregulated by inoculating AMF. *Lipase* (*114280426*) expressed over eight times higher in the T treatment than that in the C treatment. We also found four DEGs related to lipid transfer (*114312621*, *114297815*, *114317640*, and *114303154*) were upregulated by AMF inoculation.

### Key Genes Excavation and Contribution of Different Differentially Expressed Genes Categories on Root System Architecture Induced by Arbuscular Mycorrhizal Fungus

**FIGURE 5:**
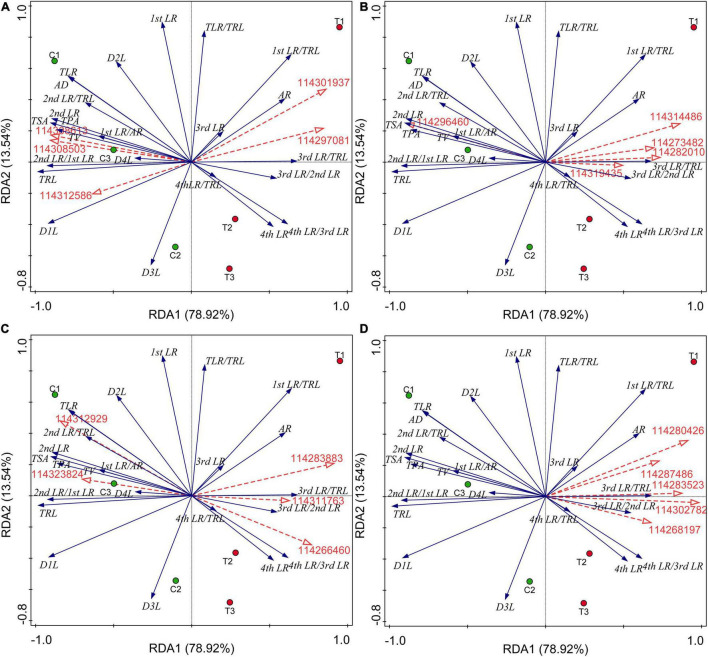
Redundancy analysis (RDA) indicating the correlations between key DEGs related to different categories and root indexes. **(A)** DEGs related to P metabolism. **(B)** DEGs related to sugar metabolism; **(C)** DEGs related to plant hormone; **(D)** DEGs related to lipid metabolism. Blue and red arrows indicated various root indexes and key DEGs induced by AMF inoculation, respectively. The annotation of DEGs, filled with yellow, was listed in Supplementary Data 2. AR, adventitious root number; LR, lateral root number; TLR, total lateral root number; TRL, total root length; TPA, total root projected area; TSA, total root surface area; TV, total root volume; AD, average diameter of root; D1L: length of 0.000 mm ≤ AD < 0.500 mm; D2L: length of 0.500 mm ≤ AD < 2.000 mm; D3L: length of 2.000 mm ≤ AD < 3.000 mm; D4L: length of 3.000 mm ≤ AD < 5.000 mm.

All the results of correlation analysis between DEGs of different categories and all root indexes were placed in Supplementary Data 4. We found that all these DEGs were not significantly correlated with the first LR number and TLR/TRL ratio. Considering a large number of DEGs, we further performed the RDA to seek out key genes regulating the RSA induced by AMF inoculation. The results were showed in Figure 5 and all potential key DEGs were filled with yellow in Supplementary Data 2. In detail, initially, as showed in Figure 5A, *114308503* (*type I inositol 1,4,5-trisphosphate 5-phosphatase 2 isoform X2*), *114308613* (*probable protein phosphatase 2C 5*), and *114312586* (*protein-tyrosine-phosphatase MKP1-like*) had positive correlations with the formation of first LR and second LR, the numbers of second LR and TLR, TSA, TPA, TRL, TV, and average diameter (AD), while *114297081* (*glycerol-3-phosphate acyltransferase 5*) and *114301937* (*1-deoxyxylulose 5-phosphate synthase*) positively correlated with AR and higher classes of LR number (third and fourth LR), which were consistent with the correlation analysis (Supplementary Data 4). In Figure 5B, *114296460* (*probable carbohydrate esterase*) significantly positively correlated with the second number, TLR number, ratio of second LR/first LR, TRL, TPA, TSA, and AD (Supplementary Data 4), same to the RDA analysis. Other four DEGs (*114319435*, *114273482*, *114314486*, and *114282010*) contributed to the higher level of branching and AR formation (Figure 5B), while in the DEGs related to plant hormone, these were *114311763* (*auxin-induced protein PCNT115-like isoform X3*), *114266460* (*auxin-responsive protein SAUR32-like*), and *114312929* (*ethylene-responsive transcription factor*), as shown in Figure 5C. *114283883* and *114323824* were involved in ethylene and ABA metabolisms, respectively, showing positive correlations with lower classes of LR and common root indexes (TPA, TRL, TV, and AD). Among the 67 lipid-related DEGs, extracted genes, namely *114302782*, *114287486*, *114283523*, *114268197*, and *114280426*, were all positively correlated with third and fourth LR development and AR number (Figure 5D and Supplementary Data 4).

To quantify the overall contributions of DEGs belonging to different categories, variation partitioning analysis was performed. The results showed that the explained fractions in descending order were lipid (19.69%), plant hormone (12.69%), P (12.41%), and sugar (9.43%) when considering with other three covariables (Table 5). And the total fraction explained was 19.72%. Intriguingly, getting rid of the covariables, we found that plant hormone-related DEGs played a negative role in RSA construction, and lipid-related DEGs showed the largest explained fractions (Table 5).

### Pathways Probing in AM Fungal Regulation on Tea Plant Root Branching

**FIGURE 6:**
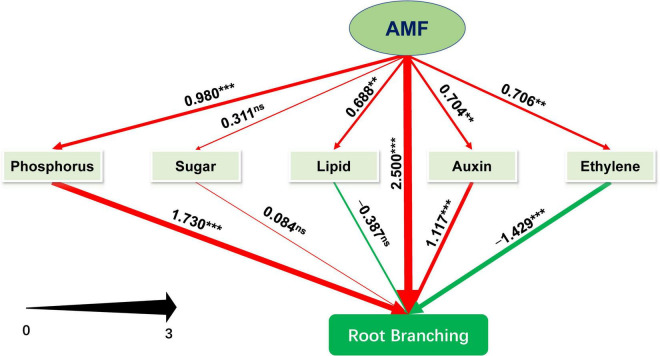
Schematic diagram of the Partial Least Squares Structural Equation Modeling (PLS-SEM). Path coefficients were showed and reflected in the width of the arrows; positive effects are shown as red arrows, negative effects as green arrows. “‘***,” “**,” and “ns” means significance at *p* < 0.001, *p* < 0.01, and non-significance, respectively.

We further performed PLS-SEM analysis to decipher how AMF inoculation affected the root branching of tea plant cutting seedlings through the metabolisms of phosphorus, sugar, plant hormones (primarily auxin and ethylene), and lipid. The model we constructed could be assumed to be acceptable according to Kock (2017). The value of Tenenhaus GoF, the widely accepted model fit index for PLS-based path modeling value (Henseler and Sarstedt, 2013), was 0.515, larger than 0.36 (for large effect size), indicating the acceptance of the model. And other related results of “Model fit and quality indices” were also presented in Supplementary Table 3. As shown in Figure 6 and Supplementary Table 4, except for sugar metabolism with non-significant promotion (0.311), inoculating with AMF significantly promoted metabolisms related to phosphorus (0.980), ethylene (0.706), lipid (0.688), and auxin (0.704), and directly enhanced root branching (2.500). Auxin- and phosphorus-related metabolism significantly facilitated the root branching, while lipid (−0.387) and ethylene (−1.429, *p* < 0.001) were negatively contributed to the root branching. In this experiment, sugar slightly promoted the root branching with no significant difference. Moreover, the indirect (1.234, *p* < 0.001) and total effect (3.374, *p* < 0.001) of AMF on root branching were both significantly positive (Supplementary Table 4). Taking together, AMF regulating root branching was mainly simultaneous through the direct pathway (or unknown ways) and indirect pathway by influencing the metabolisms of P and auxin (Figure 6).

## Discussion

The mycorrhizal colonization of roots in tea plant cutting seedlings was 26.67% in this study, similar to the results of Xia et al. (2018) and our previous studies on citrus (Chen et al., 2017a,b). The low mycorrhizal colonization may be caused by that the applied AMF was not isolated from the indigenous species, and Liu et al. (2017) found that *Septoglomus constrictum* is the most predominant AMF species in Anhui tea plant area. Berruti et al. (2013) also unraveled that the non-specific commercial AMF inocula result in poor mycorrhization in *Camellia japonica* L. Moreover, Zhu et al. (2015) found that the mycorrhizal colonization will increase with the time of the experiment, indicating the gradual adaptation of AMF to the hosts. In our study, we harvested the plants at 6 months after being treated with AMF, and the reducing effects of AMF on plant growth was significant, such as plant height and shoot biomass (Table 1), which was probably related to the low colonization and non-specific AMF species. Since tea plant can produce abundant secondary metabolites (Yue et al., 2014), among which terpenoids (monoterpenoids, diterpenoids, diterpenes, steroids, and sterols) and phenolics (tannins, flavonoids, polyphenols) were involved in defending against a variety of herbivores and microorganisms as well as various kinds of abiotic stresses (Yang et al., 2013; Zaynab et al., 2018), the mass construction of mycorrhiza symbiosis may be hindered. Additionally, the increasing sucrose content in the root (Figure 1), the promoted root branching (Table 3 and Figure 6), and the promoting effect of corresponding DEGs on RSA unraveled by RNA-seq analysis (Figure 5) may imply that the general positive effects induced by AMF could emerge when elongating the experimental time.

As the major characterization of root traits in soil, RSA plays a great important role in plant growth and development. Generally, better RSA not only elevates the absorption and utilization of water and mineral nutrients, but also becomes the basis for constructing a stable ecological community (Chen et al., 2016), thus promoting the tolerance to various abiotic stress (Leftley et al., 2021; Vázquez-Glaría et al., 2021). The response of RSA to diverse environmental conditions and relevant regulation mechanisms are always attracting the attention of numerous excellent researchers to meet healthy food production when encountering with drought, flooding, salinity, and other adverse circumstances. Affected by intrinsic genetic and environmental factors (Chen et al., 2016), such as root length, root branching, soil texture, nutrients, and water, RSA is also regulated by microorganisms like AMF (Chen et al., 2017a), endophyte (Verma et al., 2021), and rhizobia (Concha and Doerner, 2020). In our experiment, inoculation with AMF decreased the root morphogenesis overall as reflected in root biomass, TRL, TPA, TSA, and TV, which was not consistent with previous studies finding positive effects because of overall promotion of plant growth (Chen et al., 2017a; Xia et al., 2018; Wang et al., 2020). This might be on account of different AMF species, host plants, and experimental duration. The similar decreased tendency of root AD induced by AMF (Table 1) also indicated that plant growth in the future will benefit from the proportion of fine roots by enhancing the absorption of water and nutrients (Stock et al., 2021).

So far, studies on the effect of AMF on tea plant RSA are only focusing on the physiological and biochemical levels (Chen et al., 2020) and the relative profound mechanisms have not yet been reported. Therefore, we performed RNA-seq analysis in order to disclose the AM fungal regulation mechanism on RSA in the tea plant. High-quality clean reads (Table 4) and 2047 DEGs with 1171 upregulated in mycorrhizal treatment were obtained (Supplementary Data 1), which facilitated the following exploration of gaining insight into the regulatory network of RSA changes induced by AMF. The previous studies found that AMF modulates RSA by affecting phytohormones (auxin, ethylene, etc.), internal factors (LR formation, root vitality, etc.), nutrient levels (P, N, etc.), and carbohydrate allocation (Fusconi, 2014; Chen et al., 2016, 2017a). Moreover, lipid, vital for AM fungal propagation, is recently found to be only *de novo* biosynthesized in the plant but not in AMF (Jiang et al., 2017; Luginbuehl et al., 2017). Considering its crucial role in plant defense to biotic and abiotic stresses (Safi et al., 2015; Feng et al., 2020b), we also dug out the DEGs related to lipid metabolism from RNA-seq results together with other three categories of DEGs, namely metabolisms pertaining to phosphorus, sugar, and phytohormones, in order to decipher the mechanism of AM fungal regulation on RSA in tea plant cutting seedlings. In this experiment, 42, 39, 39, and 67 DEGs were found to be involved in the metabolisms of P, sugar, phytohormone, and lipid, respectively (Figure 4 and Supplementary Data 2), and the correlations of these DEGs and RSA were emphatically analyzed and discussed.

Phosphorus, an essential macronutrient for plant growth and development, has become a limiting factor for plant growth and yield because of its low inorganic phosphate availability, especially in acid red-yellow soil. Plants absorb inorganic phosphate (Pi) from the soil through their roots and transfer Pi to various organs or tissues by phosphate transporters, which are responsible for Pi uptake, translocation, and remobilization (Wang et al., 2021). Purple acid phosphatases promote root growth by effectively hydrolyzing organic phosphorus such as phosphate esters and anhydrides into inorganic phosphorus that can be directly absorbed and utilized by plants under Pi starvation (Tran et al., 2010; Deng et al., 2020). In our study, DEGs related to purple acid phosphatase (*114304683*, *114283038*, *114301217*, *114261192*) and phosphate transporter (*114261688*) were both upregulated by AMF inoculation, which might indicate the accelerated absorption of Pi from soil. The positive effect of P metabolism on RSA and root branching revealed by variation partitioning and PLS-SEM analysis, respectively, may further confirm that the promotion of growth will occur, though at present, the P content in the shoot was not increased.

In sugar metabolism, sugar transport protein-related DEGs (*114263353*, *114280123*, *114287094*, *114314873*) were all downregulated in the mycorrhizal treatment, might suggesting the competition for carbohydrates by AMF with plant roots. Sugar transport proteins are responsible for transporting hexoses, hydrolyzed from sucrose, into cells in sink tissues (Eom et al., 2015). Taken together, this may explain that the significantly increased sucrose in the root by inoculation AMF did not improve the overall root development (Table 1), as well as the non-significant improvement in root branching (Figure 6), except for the promotion of RSA by sugar metabolism (Table 5).

The lipid in mycorrhizal symbiosis has recently gained more and more attention (Drijber and Jeske, 2019; Feng et al., 2020a,b; Gargouri et al., 2021; Liu et al., 2021b) because of its indispensable role in AM fungal propagation as important as sugar (Jiang et al., 2017; Luginbuehl et al., 2017). The previous studies show that *Arbuscular Mycorrhization 2* (*RAM2*), encoding a glycerol-3-phosphate acyltransferase, is necessary for transferring lipids from plants to AMF and is also likely to play a “signaling” role at the root surface (Jiang et al., 2017; Liu et al., 2021b). Two *Stunted Arbuscule* genes, *STR* and *STR2*, localized in the periarbuscular membrane, encode ATP-binding cassette transporters, which is also responsible for lipid transfer to AMF and indispensable for arbuscule formation (Zhang et al., 2010; Gutjahr et al., 2012; Jiang et al., 2017). In our study, homologous genes of *RAM2* (*114297081*), *STR* (*114323273*), and *STR2* (*114276941*) were all upregulated in the mycorrhizal treatment (Supplementary Data 1), indicating a successful mycorrhizal symbiosis. Moreover, 67 DEGs pertaining to lipid metabolism were found in our experiment, and screened key genes, including *RAM2*, were both positively related to higher classes of LR formation (third and fourth LR) and the density of the first LR unveiled by RDA analysis in Figure 5. But in PLS-SEM results (Figure 6), the factor of lipid metabolism slightly negatively affected the root branching of tea plant. These probably underlined that the AMF improved the root branching of higher LR through lipid pathway was not for promoting plant growth but for increasing inoculation in the future.

With respect to phytohormones, auxin plays a critical role in AR and LR formations (Alaguero Cordovilla et al., 2021), which determines the RSA to a great extent. In our study, 13 auxin-related DEGs were all upregulated (Supplementary Data 2) and significantly positive promotion of root branching was found by the PLS-SEM analysis (Figure 6), indicating the important role involved in AMF modulating RSA. Previous studies showed that sugar and P, both can modulate auxin metabolism to induce LR formation (Sairanen et al., 2012; Talboys et al., 2014). Sugar signal needs time to be transferred to auxin, which could partially account for the non-significant effect of sugar metabolism induced by AMF on root branching (Figure 6). Overall, auxin was extraordinarily important in AM fungal regulation on tea plant RSA construction. Another key plant hormone was ethylene, generally acting as a negative regulator of LR formation (Singh et al., 2015), which is consistent with our current (Figure 6) and previous studies (Chen et al., 2017a,b) on citrus. Moreover, ethylene also regulates root growth through the auxin pathway in response to sugar and phosphate deficiency (Růzicka et al., 2007; Roldan et al., 2013; Zhang et al., 2016), indicating the complex regulation network of AMF modulating RSA. Besides, ABA is involved in plant stress responses (Kumar et al., 2019) and could modulate root patterns induced by AMF (Song et al., 2020; Zhang et al., 2020). Similar results were found in this study that five ABA-related DEGs were significantly affected by AMF inoculation. Nevertheless, other phytohormones in tea plant roots did not strongly susceptible to inoculating AMF in this study.

## Conclusion

An AMF species, *Rhizophagus intraradices*, was inoculated with tea plant cutting seedlings to obtain new insights into the mechanism of the AM fungal regulation on RSA with focusing on root branching *via* pathways of P metabolism, sugar metabolism, plant hormones, and lipid metabolism. In our experiment, AMF inoculation mainly strongly enhanced the higher classes of LR formation and AR number, and significantly increased the root sucrose content, whereas decreased the root biomass, as well as the shoot biomass and P content in it. Further RNA-seq results disclosed that AMF altered RSA mainly through positive regulations on root branching by direct or indirect pathways (P- and auxin-related metabolisms), despite the declining of total root biomass. In general, our study provided a global view of the underlying molecular mechanisms about the AM fungal regulation on RSA in tea plant cutting seedlings.

## Data Availability Statement

The original contributions presented in the study are publicly available. This data can be found here: National Center for Biotechnology Information (NCBI) BioProject database under accession number PRJNA763027.

## Author Contributions

WC and JZ conceived and designed the manuscript. WC wrote the manuscript. TY, TN, JZ, and QS reviewed and edited the manuscript. All authors approved the manuscript for submission.

## Conflict of Interest

The authors declare that the research was conducted in the absence of any commercial or financial relationships that could be construed as a potential conflict of interest.

## Publisher’s Note

All claims expressed in this article are solely those of the authors and do not necessarily represent those of their affiliated organizations, or those of the publisher, the editors and the reviewers. Any product that may be evaluated in this article, or claim that may be made by its manufacturer, is not guaranteed or endorsed by the publisher.

## Footnotes

1
http://tpdb.shengxin.ren/

2
http://www.genome.jp/kegg/

3
http://kobas.cbi.pku.edu.cn/

4
http://www.ncbi.nlm.nih.gov/sra
