# Supplementary material for: Arbuscular Mycorrhizal Fungus Alters Root System Architecture in Camellia sinensis L. as Revealed by RNA-Seq Analysis
Source: Front Plant Sci. 2021 Nov 12;12:777357. doi: 10.3389/fpls.2021.777357 (PMC8636117; doi:10.3389/fpls.2021.777357)

Supplementary Material

Title:

Arbuscular mycorrhizal fungus alters root system architecture in *Camellia sinensis* L. as revealed by RNA-Seq analysis

Authors:

Weili Chen, Tao Ye, Qinyu Sun, Tingting Niu, Jiaxia Zhang*

*****Correspondence: Jiaxia Zhang: [zhangjiaxia035@163.com](mailto:zhangjiaxia035@163.com)

## Supplementary Tables and Figures

Supplementary Table 1 Primers of each gene in real-time quantitative RT-PCR

| **Gene ID** | **Gene Description** | **Sequence of primer (5’-3’)** |
| --- | --- | --- |
| 114278857 | auxin-responsive protein SAUR32-like | F: ACCCGAAGGTTGCTTCTCAG  R: GCCACAGCGACCCATAGAAA |
| 114308158 | auxin response factor 10 | F: ACACACCGGAGATGAACCCT  R: GAAGCAGGTTTCTCCCACAAC |
| 114277140 | auxin-repressed protein | F: GGACGATGTGTTAGCCGGAC  R: CGACGACGAAGACGAACTTG |
| 114282010 | serine carboxypeptidase-like 40 | F: CCACACGGTGAATCAACCAA  R: AAGAAGGAAGGCTAGGATCGG |
| 114301937 | 1-deoxyxylulose 5-phosphate synthase | F: CACACCTGACAACATCCCCA  R: GGTGTGGGTTGCTTTTCTCC |
| 114263278 | NADPH-dependent 1-acyldihydroxyacetone phosphate reductase | F: GGCTAAGGCTGGTCCAACAA  R: ACTAGCGCCACGTTAATCCC |
| 114288835 | terpenoid synthase, partial | F: GGTTTCGACTCGCTTTCGTC  R: AAGACGTGTCAACCACCCATT |
| 114273524 | lactate dehydrogenase | F: AATAGCCAAGGTTGAGAGAAGAGT  R: CCTAGTCCATTAGGGTTGCTTTTC |
| 114305402 | gibberellin-regulated family protein | F: ATTGCGTGGAAGCTCTCTGG  R: CAGGAAGCTCAAGACTGGGA |
| 114260196 | ethylene response factor | F: CCATCCCGACTCTGTCTTCTC  R: GGGCTCTCTGCTCCACTCAT |
| 114323882 | ethylene-responsive transcription factor ERF110 | F: TCCCTCTCCACCCAATACCC  R: CCGAACCCCTCTGTAGGACT |
| 114263353 | sugar transport protein 1-like | F: CAAGTTCAGAGGCAACAAGGC  R: TCTGTGCTCATCCTCTGCAAC |
| 114278857 | auxin-responsive protein SAUR32-like | F: CTTCGTCATCTTCTTCGTGCC  R: TCCATTCTTCATCTCGATTTCCTT |
| AB120309.1 | Camellia sinensis 18S Ribosomal RNA | F: CGCGCAAATTACCCAATCCT  R: ACCAGACTTGCCCTCCAATG |

Supplementary Table 2 Correlation analysis results between contents of sugar and phosphorus and indexes of root system architecture. Correlation coefficients filled with red and dark red indicated significance at *p* < 0.05 and *p* < 0.01, respectively. AR: adventitious root number; LR: lateral root number; TLR: total lateral root number; TRL: total root length; TPA: total root projected area; TSA: total root surface area; TV: total root volume; AD: average diameter of root; D1L: length of 0.000 mm ≤ AD < 0.500 mm; D2L: length of 0.500 mm ≤ AD < 2.000 mm; D3L: length of 2.000 mm ≤ AD < 3.000 mm; D4L: length of 3.000 mm ≤ AD < 5.000 mm.

| RSA indexes | Sucrose | Reducing sugar | Soluble sugar | Phosphorus |
| --- | --- | --- | --- | --- |
| TRL | **-0.813*** | -0.549 | -0.461 | 0.638 |
| TPA | -0.705 | -0.491 | -0.589 | 0.509 |
| TSA | -0.705 | -0.491 | -0.589 | 0.509 |
| TV | -0.65 | -0.566 | -0.639 | 0.439 |
| AD | -0.729 | -0.271 | -0.505 | 0.654 |
| D1L | **-0.833*** | -0.491 | -0.217 | 0.714 |
| D2L | -0.252 | -0.276 | -0.623 | 0.082 |
| D3L | -0.197 | -0.627 | -0.186 | 0.195 |
| D4L | -0.513 | 0.301 | 0.283 | 0.537 |
| AR | 0.709 | 0.68 | 0.301 | -0.787 |
| 1^st^ LR | -0.329 | -0.178 | -0.544 | 0.469 |
| 2^nd^ LR | **-0.957**** | -0.659 | -0.661 | **0.941**** |
| 3^rd^ LR | 0.541 | 0.66 | 0.383 | -0.656 |
| 4^th^ LR | 0.800 | 0.683 | 0.715 | **-0.892*** |
| TLR | **-0.818*** | -0.488 | -0.658 | **0.820*** |
| 1^st^ LR / AR | -0.763 | -0.666 | -0.553 | **0.919**** |
| 2^nd^ LR / 1^st^ LR | **-0.982**** | -0.734 | -0.582 | **0.926**** |
| 3^rd^ LR / 2^nd^ LR | 0.811 | 0.772 | 0.624 | **-0.919**** |
| 4^th^ LR / 3^rd^ LR | **0.860*** | 0.673 | 0.723 | **-0.876*** |
| 1^st^ LR / TRL | 0.423 | 0.294 | -0.045 | -0.207 |
| 2^nd^ LR / TRL | **-0.877*** | -0.609 | -0.635 | **0.961**** |
| 3^rd^ LR / TRL | **0.896*** | 0.791 | 0.602 | **-0.942**** |
| 4^th^ LR / TRL | **0.845*** | 0.705 | 0.709 | **-0.921**** |
| TLR / TRL | -0.186 | -0.053 | -0.405 | 0.371 |

Supplementary Table 3 Model fit and quality indices

| **Index** | **Value** | **Value Interpretation** |
| --- | --- | --- |
| Average path coefficient (APC) | 0.967  *p* < 0.001 | Significant if *p* <  0.05 |
| R-squared (AARS) | 0.844  *p* < 0.001 | Significant if *p* <  0.05 |
| Average adjusted R-squared (AARS) | -0.797  *p* < 0.001 | Significant if *p* <  0.05 |
| Average block VIF (AVIF) | Inf | acceptable if <= 5, ideally <= 3.3 |
| Average full collinearity VIF (AFVIF) | Inf | acceptable if <= 5, ideally <= 3.3 |
| Tenenhaus GoF (GoF) | 0.515 | small >= 0.1, medium >= 0.25, large >= 0.36 |
| Sympson’s paradox ratio (SPR) | 0.818 | acceptable if >= 0.7, ideally = 1 |
| Statistical suppression ratio (SSR) | 0.727 | acceptable if >= 0.7 |
| Nonlinear bivariate causality direction ratio (NLBCDR) | 0.727 | acceptable if >= 0.7 |

Supplementary Table 4 Total effects of different paths.

| **Path** | **Path coefficient** | ***p* value** |
| --- | --- | --- |
| AMF→P | 0.980 | <0.001 |
| AMF→Sugar | 0.311 | 0.166 |
| AMF→Lipid | 0.688 | 0.008 |
| AMF→Auxin | 0.704 | 0.007 |
| AMF→Ethylene | 0.706 | 0.006 |
| AMF→Root branching (indirect) | 1.234 | <0.001 |
| AMF→Root branching(direct) | 2.500 | <0.001 |
| AMF→Root branching | 3.734 | <0.001 |
| P→Root branching | 1.730 | <0.001 |
| Sugar→Root branching | 0.084 | 0.415 |
| Lipid→Root branching | -0.387 | 0.103 |
| Auxin→Root branching | 1.117 | <0.001 |
| Ethylene→Root branching | -1.429 | <0.001 |

Supplementary Figure 1 Validation of RNA-seq results by qRT-PCR. Data from qRT-PCR (Black-filled circle) and RNA-Seq (White column) of 12 selected genes were means of three replicates and bars represent Standard Error. C: non-mycorrhizal treatment, T: mycorrhizal treatment.


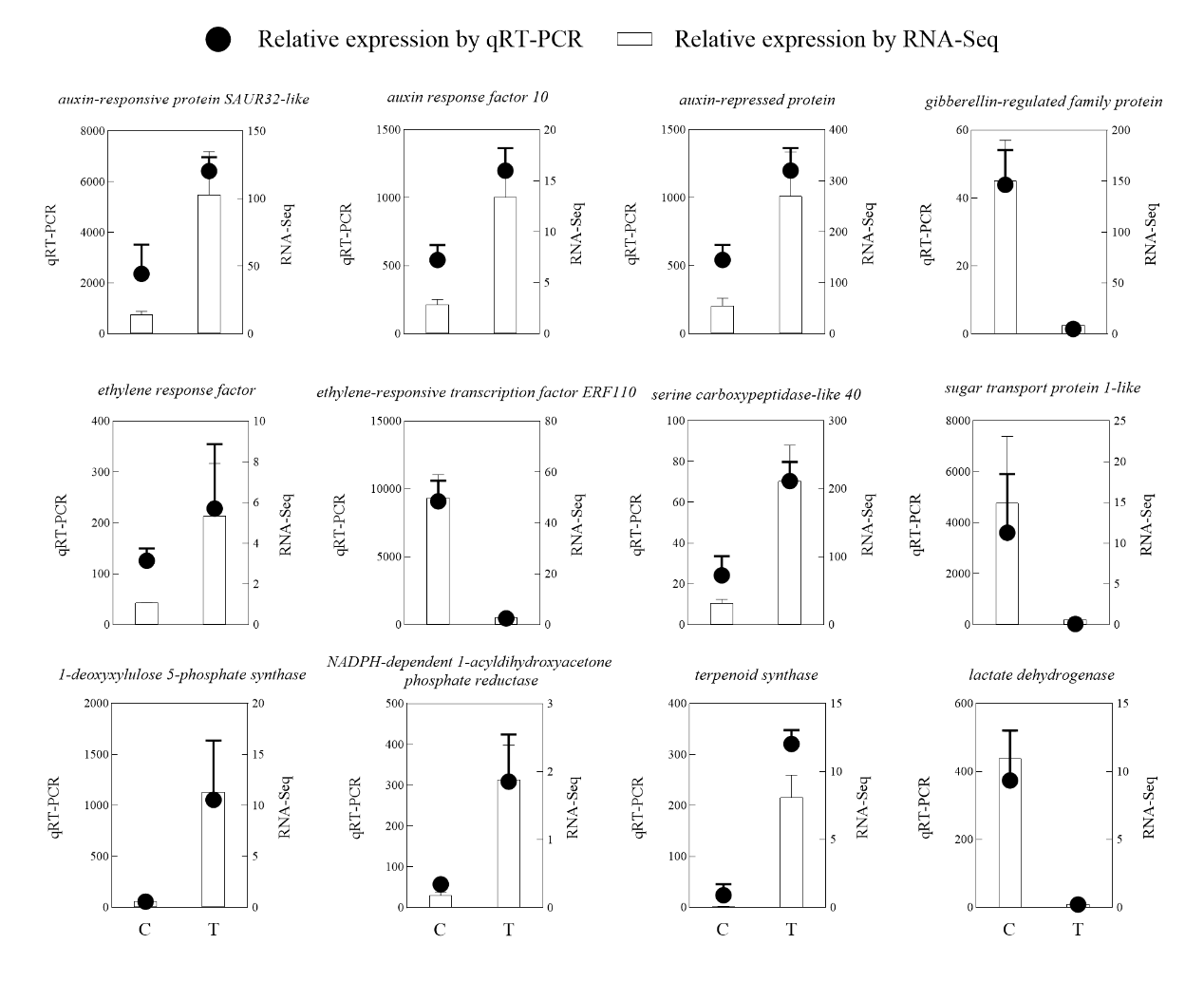


Supplementary Figure 2 Correlation analysis of fold change data between qRT-PCR and RNA-seq. Scatterplots were generated by the log2(Fold change) from RNA-seq (x-axis) and qRT-PCR (y-axis).


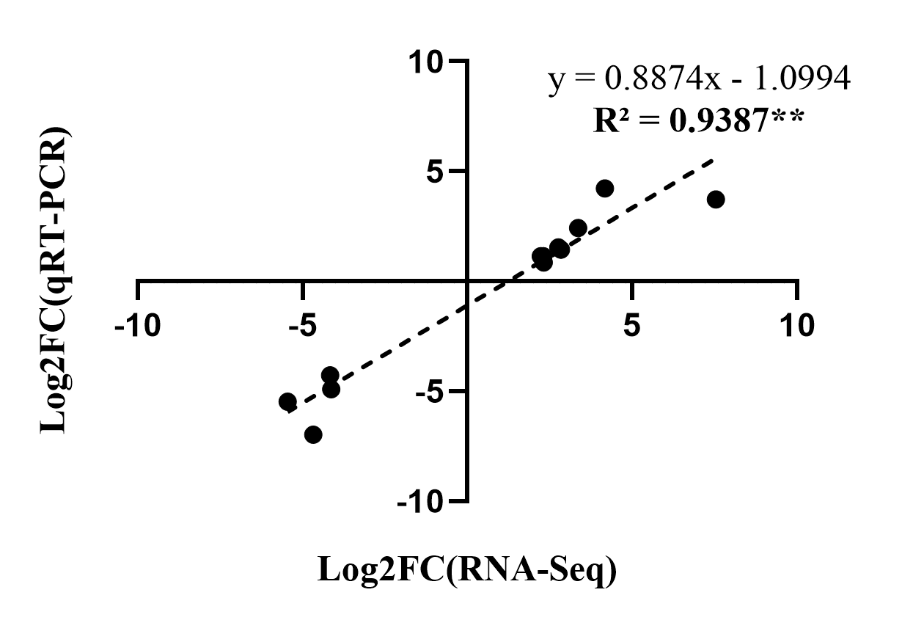


Supplementary Figure 3 Number of differentially expressed genes enriched in different GO terms


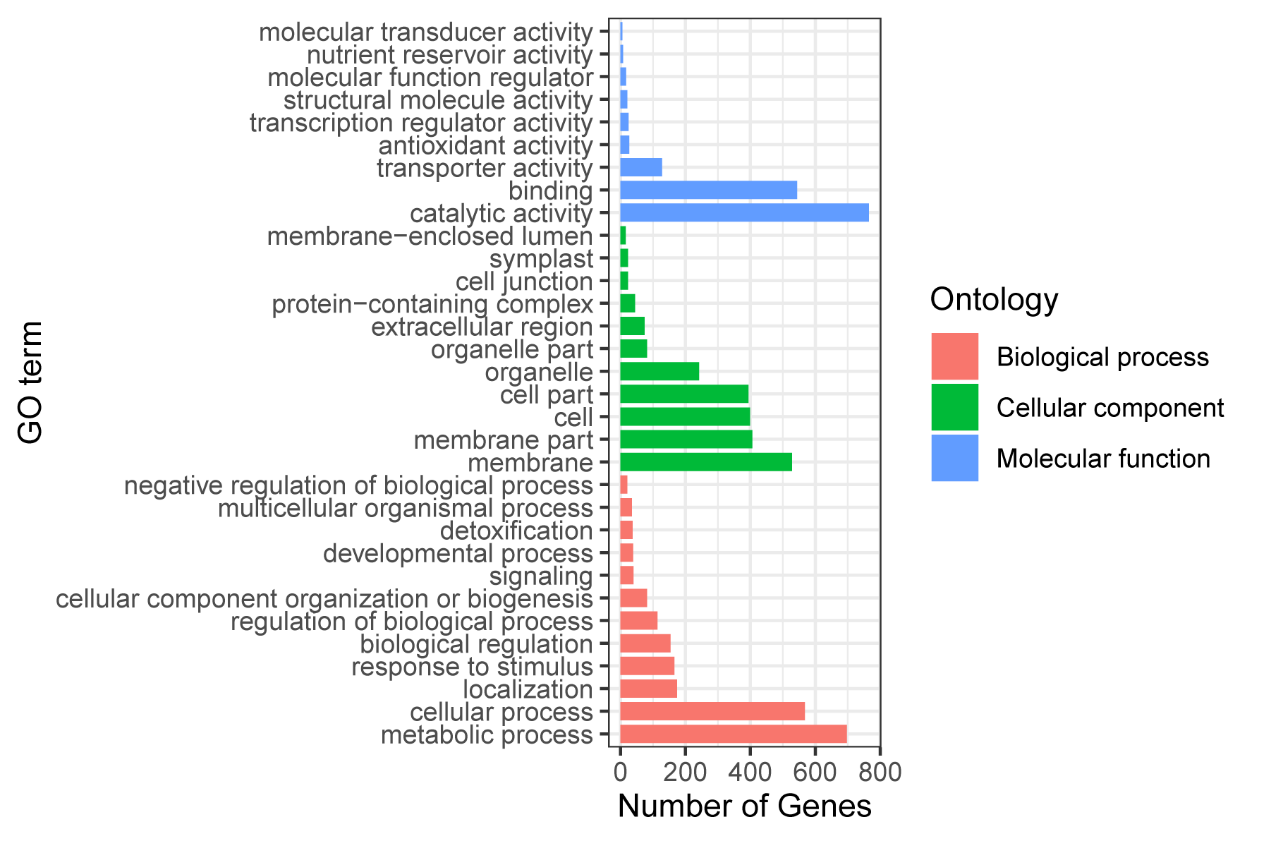


Supplementary Figure 4 The main results of KEGG pathway analysis


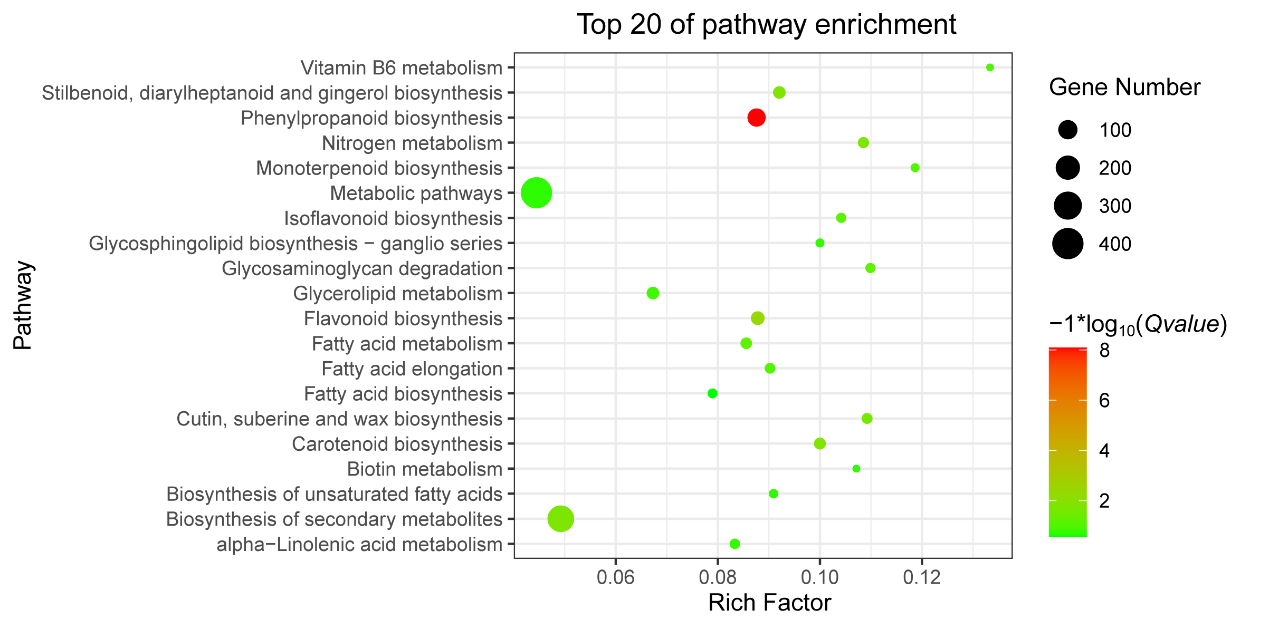

Supplement: Supplementary file 5 [file Data_Sheet_5.docx]
